# The different manifestations of COVID-19 in adults and children: a cohort study in an intensive care unit

**DOI:** 10.1186/s12879-021-05786-5

**Published:** 2021-01-20

**Authors:** Mònica Girona-Alarcon, Sara Bobillo-Perez, Anna Sole-Ribalta, Lluisa Hernandez, Carmina Guitart, Ricardo Suarez, Mònica Balaguer, Francisco-Jose Cambra, Iolanda Jordan, on behalf of the KIDS-Corona study group, Kids Corona Platform

**Affiliations:** 1 Paediatric Intensive Care Unit, Hospital Sant Joan de Déu, University of Barcelona, Barcelona, Spain; 2 Immunological and Respiratory Disorders in the Paediatric Critical Patient Research Group, Institut de Recerca Hospital Sant Joan de Déu, Hospital Sant Joan de Déu, Barcelona, Spain; 3 Emergency Transport System, Hospital Sant Joan de Déu, Barcelona, Spain; 4 Paediatric Intensive Care Unit, CIBERESP, Hospital Sant Joan de Déu, University of Barcelona, Barcelona, Spain

**Keywords:** SARS-CoV-2, COVID-19, Acute respiratory distress syndrome, Multisystem inflammatory syndrome, Biomarkers

## Abstract

**Background:**

The severe acute respiratory syndrome coronavirus 2 (SARS-CoV-2) has collapsed health systems worldwide. In adults, the virus causes severe acute respiratory distress syndrome (ARDS), while in children the disease seems to be milder, although a severe multisystem inflammatory syndrome (MIS-C) has been described. The aim was to describe and compare the characteristics of the severe COVID-19 disease in adults and children.

**Methods:**

This prospective observational cohort study included the young adults and children infected with SARS-CoV-2 between March–June 2020 and admitted to the paediatric intensive care unit. The two populations were analysed and compared focusing on their clinical and analytical characteristics and outcomes.

**Results:**

Twenty patients were included. There were 16 adults (80%) and 4 children (20%). No mortality was recorded.

All the adults were admitted due to ARDS. The median age was 32 years (IQR 23.3–41.5) and the most relevant previous pathology was obesity (*n* = 7, 43.7%). Thirteen (81.3%) needed mechanical ventilation, with a median PEEP of 13 (IQR 10.5–14.5). Six (37.5%) needed inotropic support due to the sedation. Eight (50%) developed a healthcare-associated infection, the most frequent of which was central line-associated bloodstream infection (*n* = 7, 71.4%). One patient developed a partial pulmonary thromboembolism, despite him being treated with heparin.

All the children were admitted due to MIS-C. Two (50%) required mechanical ventilation. All needed inotropic support, with a median vasoactive-inotropic score of 27.5 (IQR 17.5–30).

The difference in the inotropic requirements between the two populations was statistically significant (37.5% vs. 100%, *p* < 0.001). The biomarker values were higher in children than in adults: mid-regional pro-adrenomedullin 1.72 vs. 0.78 nmol/L (*p* = 0.017), procalcitonin 5.7 vs. 0.19 ng/mL (*p* = 0.023), and C-reactive protein 328.2 vs. 146.9 mg/L (*p* = 0.005). N-terminal pro-B-type natriuretic peptide and troponins were higher in children than in adults (*p* = 0.034 and *p* = 0.039, respectively).

**Conclusions:**

Adults and children had different clinical manifestations. Adults developed severe ARDS requiring increased respiratory support, whereas children presented MIS-C with greater inotropic requirements. Biomarkers could be helpful in identifying susceptible patients, since they might change depending on the clinical features.

## Background

The novel severe acute respiratory syndrome coronavirus 2 (SARS-CoV-2), which first appeared in December 2019 in Wuhan, has caused the coronavirus (COVID-19) related pandemic and has been declared a significant threat to international health by the World Health Organization. As of 20 December 2020, 75 million cases have been reported across the globe, causing more than 1,6 million deaths [1]. It has collapsed health systems worldwide, mainly due to its respiratory manifestations in the adult population, causing many cases of acute respiratory distress syndrome (ARDS) [2].

Interestingly, not all patients have the same manifestations; while some of them have hypoxemia due to pneumonia with a ventilation/perfusion mismatch (pulmonary vasoconstriction), others have this due to lower compliance and severe ARDS [3]. By contrast, in children the disease seems to be milder, with clinical symptoms similar to other acute viral respiratory infections, with a low mortality rate of around 0–0.2% [4, 5]. There are many hypothesis regarding why children are less affected by the disease. One possibility is that the lower number of angiotensin-converting enzyme 2 (ACE2) receptors in children’s nasal epithelium limits virus entry, and the higher numbers of ACE2 receptors in their lungs could act as a protective factor [6]. Recently, some reports have described paediatric patients with a multisystem inflammatory syndrome (MIS-C) that shares similarities with Kawasaki disease and toxic shock syndrome and can potentially lead to multiorgan failure and shock [7]. Biomarkers related to the cytokine storm, since they seem to have a role in detecting severely affected patients in other pathologies like sepsis [8], might be useful to distinguish between the two clearly different clinical manifestations.

Both ARDS and MIS-C are most likely post-infectious manifestations. Little is known about the specific immunity response after a SARS-CoV-2 infection. It seems that there is an initial peak viral load (at which point nasopharyngeal PCR samples can test positive for the viral RNA), and then this declines as antibodies (IgM and IgG) increase within days to weeks of symptom onset, just when inflammatory signs appear [9]. However, both the durability of the neutralising antibodies and whether there is persistent immunity against reinfection is unclear [10].

Since the clinical presentation of the severe forms of the disease implies a cytokine release syndrome with an increase in interleukin-6 (IL-6), tocilizumab has been pointed to as a useful treatment to decrease the inflammatory response [11]. In children there is no evidence regarding specific treatments, since most studies involve the adult population [4].

During the COVID-19 pandemic, both adult and paediatric patients were admitted to our paediatric intensive care unit (ICU), and a different clinical presentation and evolution were detected in each group. The aim of this study was to describe the characteristics of the disease in each specific population and to analyse the differences between adults and children regarding the clinical manifestations and analytical data.

## Methods

This was a prospective and observational cohort study, performed in the paediatric ICU of Hospital Sant Joan de Déu in Barcelona, which is a paediatric tertiary referral hospital. This paediatric ICU has 28 ventilator-beds and sees 1200 admissions per year. All the patients infected with SARS-CoV-2 (with a positive PCR result from a nasopharyngeal swab and/or positive IgM or IgG) admitted to the paediatric ICU between March and June 2020 were included, both adults and children. These two populations were analysed separately in order to compare their clinical characteristics and outcomes.

### Variables

The following demographic data were collected: age, gender, comorbidities, Paediatric Risk of Mortality Score (PRISM III) at admission (11), and reason for admission (respiratory or cardiovascular failure). Respiratory support was also analysed: positive end-expiratory pressure (PEEP), ventilator and oxygenation requirements (prone position, recruitment manoeuvres), and length of mechanical ventilation (MV) and non-invasive ventilation (NIV). Haemodynamic support requirements were analysed using the vasoactive inotropic score (VIS) (11) and the length of the inotropic treatment. The need for heparin treatment was analysed: a high heparin dose was considered as 1 mg/kg/12 h and a low heparin dose was considered as 0.5 mg/kg/12 h. The microbiological data collected included microorganisms isolated in the blood, respiratory tract, and urine cultures. Antibiotic requirements and lengths of treatment were recorded. The analytical biomarkers recorded were C-reactive protein, procalcitonin, mid-regional pro-adrenomedullin (MR-proADM), ferritin, D-dimer, lactate dehydrogenase, and blood counts. N-terminal pro-B-type natriuretic peptide (NT-proBNP) and troponins were analysed in order to assess cardiac dysfunction.

Serum levels of procalcitonin and MR-proADM were determined by immunofluorescense by means of a Time-Resolved Amplified Cryptate Emission (TRACE) and a Kryptor analyser (B·R·A·H·M·S-Diagnostica GmbH, Henningsdorf, Germany). C-reactive protein was obtained using the immunoturbidimetric assay (COBAS INTEGRA ROCHE).

The length of stay in the hospital and in the paediatric ICU, as well as the mortality rate, were also recorded.

The patients were classified, depending on their clinical presentation, as having:

- **ARDS,** identified following the Berlin Definition, specifically, respiratory failure with bilateral pulmonary opacities with PaO_2_ < 300 mmHg and PEEP or CPAP > 5 cmH_2_O [12].
- **MIS-C in children and adolescents**, identified according to the preliminary definition provided by the World Health Organization, which specifies the presence of shock, with elevated biomarkers and a positive COVID-19 test, without other microbial causes of inflammation [7]. These patients also met the CDC criteria for MIS-C: an individual aged under 21 years with fever, laboratory evidence of inflammation, and evidence of clinically severe illness requiring hospitalisation, with multisystem organ involvement AND no alternative plausible diagnosis AND positive for current or recent SARS-CoV-2 infection [13].

### Statistical analysis

A statistical analysis was performed using the IBM SPSS 25.0 Statistics® programme. Categorical variables were indicated as frequency (n) and percentage (%), whereas continuous variables were summarised as median and interquartile range (IQR) because they did not follow a normal distribution, according to the Shapiro-Wilk test. The comparison of categorical variables was performed using the χ2-test or Fisher’s exact test. Continuous variables were compared using the Mann-Whitney U test. Probability values of less than 0.05 were considered statistically significant.

## Results

During the study, 20 patients were included. There were 16 adults (80%) and 4 children (20%). All the patients had a positive PCR result from the nasopharyngeal sample except one child, who had suggestive clinical features and a positive IgG. No patient needed extracorporeal membrane oxygenation support and no deaths were recorded.

### Description of the adult patients

The 16 adults all presented with ARDS. The median age was 32 years (IQR 23.3–41.5), and 11 (68.8%) had a relevant previous pathology, the most frequent of which was obesity (*n* = 7, 43.7%). Among them, 13 (81.3%) needed MV, with a median maximum PEEP of 13 (IQR 10.5–14.5). Prone position was required in 10 patients (76.9%) and recruitment manoeuvres in 5 patients (38.5%). Six patients (37.5%) required inotropic support, in all cases due to sedation. All the patients received treatment with heparin and 6 of them (37.5%) were on high doses. The specific treatment related to COVID-19 is detailed in Table 1.

**Table 1:** Patient characteristics, risk factors, and outcomes

	Total ***n*** = 20	Adults ***n*** = 16	Children ***n*** = 4	***p***-value
**Males, n (%)**	11 (55)	9 (56.3)	2 (50)	1.000
**Age (years)**	29.5 (20.3–40)	32 (23.3–41.5)	13.5 (5.5–16.5)	**< 0.001**
**Risk factors**	11 (55)	11 (68.8)	0 (0)	**0.026**
Obesity	7 (35)	7 (43.8)		
Asthma	5 (25)	5 (31.3)		
Arterial hypertension	2 (10)	2 (12.5)		
Diabetes	1 (5)	1 (6.3)		
**Days of symptoms until admission**	9.5 (7–11.8)	10.5 (8–12)	4 (2.3–6.5)	**0.002**
**Reason for admission, n (%)**				**< 0.001**
Respiratory problems	16 (80)	16 (100)	0 (0)	
Haemodynamic problems	4 (20)	0 (0)	4 (100)	
**PRISM III**	6 (2.8–10)	6 (2.8–9.5)	8.5 (1.3–15.8)	0.567
**Respiratory support**				
Mechanical ventilation, n (%)	15 (75)	13 (81.3)	2 (50)	0.249
- Maximum PEEP (cmH_2_O)	12 (10–14)	13 (10.5–14.5)	6 (6–8)	**0.014**
- FiO2 (%)	50 (40–60)	50 (40–60)	35 (26.3–43.7)	**0.032**
- Prone position, n (%)	10 (66.7)	10 (76.9)	0 (0)	**0.036**
- Recruitment manoeuvres, n (%)	5 (33.3)	5 (38.5)	0 (0)	0.509
- Length of MV (days)	10 (4–12)	11 (8–12)	3.5 (3–4)	0.074
Non-invasive ventilation, n (%)	7 (35)	5 (31.3)	2 (50)	0.587
- CPAP, n (%)	1 (14.3)	1 (20)	0 (0)	0.495
- BiPAP, n (%)	6 (85.7)	4 (80)	2 (100)	0.495
- Length of NIV (days)	2 (2–3)	2 (1.5–3.5)	2 (2–3)	0.669
**Haemodynamic support**				
Inotropic treatment, n (%)	10 (50)	6 (37.5)	4 (100)	0.087
Inotropic due to shock, n (%)	4 (20)	0 (0)	4 (100)	**0.005**
Inotropic support length (days)	2 (1–4)	1.5 (1–2)	3 (2–4.7)	0.094
Maximum VIS	10 (5–26.3)	5 (5–10)	27.5 (17.5–30)	**0.008**
**Antithrombotic prophylaxis**				
Heparin treatment, n (%)	19 (95)	16 (100)	3 (75)	0.200
High doses, n (%)	7 (36.8)	6 (37.5)	1 (33.3)	1.000
**Specific treatment**				
Azithromycin, n (%)	17 (85)	14 (87.5)	3 (75)	0.509
Hydroxychloroquine, n (%)	18 (20)	16 (100)	2 (50)	**0.032**
Corticosteroids, n (%)	11 (55)	7 (43.8)	4 (100)	0.094
Tocilizumab, n (%)	10 (50)	9 (56.3)	1 (25)	0.582
Interferon, n (%)	5 (25)	5 (31.3)	0 (0)	0.530
**Antibiotic treatment**				
Antibiotic requirement, n (%)	17 (85)	13 (81.3)	4 (100)	1.000
Length of antibiotic treatment (days)	7 (6.5–8.5)	7 (7–9)	6.5 (5.3–7)	0.135
**Outcomes**				
Length of stay in paediatric ICU (days)	10 (5–16.3)	12 (5.7–17)	6 (4.3–7)	0.064
Length of stay in hospital (days)	18 (12.5–29.3)	21 (13.7–29.8)	12 (11–13)	0.170
Pulmonary thromboembolism, n (%)	1 (5)	1 (6.3)	0 (0)	1.000
Need for ECMO, n (%)	0 (0)	0 (0)	0 (0)	
Mortality, n (%)	0 (0)	0 (0)	0 (0)	

Values are expressed as frequency (percentage) for qualitative variables and as median (interquartile range) for quantitative variables. n: number of patients. *p*-value: significant value. *PRISM III* Paediatric Risk of Mortality III, *VIS* vasoactive inotropic score, *MV* mechanical ventilation, *NIV* non-invasive ventilation, *ICU* Intensive Care Unit, *ECMO* extracorporeal membrane oxygenation

Eight patients (50%) developed a healthcare-associated infection, and the most frequent of them were central line-associated bloodstream infections (*n* = 7, 43.8%) The microbiological details are summarised in Table 2.

**Table 2:** Blood parameters and microbiological data

	Total ***n =*** 20	Adults ***n =*** 16	Children ***n =*** 4	***p-***value
**Biomarkers**				
Mid-regional pro-adrenomedullin (nmol/L)	0.85 (0.65–1.15)	0.78 (0.64–0.88)	1.72 (1.55–2.77)	**0.017**
Procalcitonin (ng/mL)	0.22 (0.12–0.48)	0.19 (0.11–0.37)	5.7 (1.3–7.77)	**0.023**
C-reactive protein (mg/L)	181.5 (62.5–237.7)	146.95 (57.58–192.98)	328.2 (252.8–351.15)	**0.005**
Troponines (ng/mL)	0.004 (0.002–0.035)	0.003 (0.002–0.014)	0.09 (0.01–2.85)	0.039
NT-proBNP	2414 (59–4840)	59 (55.5–375)	4057 (2629–18,211)	0.034
**Blood samples**				
Lymphocytes (/mm3)	1000 (700–1175)	1000 (700–1100)	1100 (475–8175)	0.703
Prothrombin time (%)	84.5 (65.6–94.8)	88 (83.3–96.8)	56.4 (47.5–63.6)	**0.006**
Triglycerides (mg/dL)	391 (180.5–603.5)	413 (255–623)	158 (116–167)	**0.015**
D-dimer, at admission (mg/L)	2.45 (1.1–5)	2.45 (0.82–4.46)	3.94 (1.35–7)	0.571
D-dimer, maximum (mg/L)	6.3 (2.1–8.6)	5.45 (1.89–9.98)	6.75 (5.63–8.33)	0.539
Ferritin, maximum (ug/L)	910 (359.7–3614.5)	999.5 (311.1–4210.5)	714.8 (567–1369.25)	0.637
Lactate dehydrogenase, maximum (IU/L)	838 (760–1464)	1053.5 (711–1616)	800 (791–819)	0.576
IL-6, maximum (pg/mL)	460.5 (52.5–1000)	709.5 (24.7–1000)	141.75 (61.1–804.75)	0.829
**Microbiological data**				
Healthcare-associated infection, n (%)	8 (40)	8 (50)	0 (0)	0.103
CLABSI, n (%)	7 (35)	7 (43.8)		
- *S. epidermidis*	5 (71.4)	5 (71.4)		
- *K. pneumoniae*	1 (14.3)	1 (14.3)		
- *C. albicans*	1 (14.3)	1 (14.3)		
CAUTI, n (%)	5 (25)	5 (31.3)		
- *P. aeruginosa*	2 (40)	2 (40)		
- *K. pneumoniae*	1 (10)	1 (10)		
- *E. coli*	2 (40)	2 (40)		
VAP, n (%)	2 (10)	2 (40)		
- *R. ornithinolytica*	1 (50)	1 (50)		
- *P. aeruginosa*	1 (50)	1 (50)		

Values are expressed as frequency (percentage) for qualitative variables and as median (interquartile range) for quantitative variables. n: number of patients. *p*-value: significant value. *IL-6* interleukin-6, *CLABSI* central line-associated bloodstream infection, *CAUTI* catheter-associated urinary tract infection, *VAP* ventilator-associated pneumonia

On the blood test, the patients presented low lymphocyte count, high triglycerides levels, and a high D-dimer values during the clinical evolution. The median levels for IL-6 were high. The median for MR-proADM was 0.78 nmol/L (IQR 0.64–0.88), for procalcitonin it was 0.19 ng/mL (IQR 0.11–0.37), and for C-reactive protein it was 146.95 mg/L (IQR 57.6–193). The detailed results of the blood sample tests are included in Table 2.

The adult patients had a median length of stay in the ICU of 12 days (IQR 5.7–17), and they had a total length of stay in the hospital of 21 days (IQR 13.7–29.8).

### Description of the paediatric patients

Four children were admitted during the study period. None of them had a pre-existing disease or risk factor. All presented with MIS-C and required admission to the paediatric ICU due to shock. Two of them (50%) required MV, with low ventilator parameters, and two (50%) required NIV. All the patients needed inotropic support due to the shock, with a median VIS of 27.5 (IQR 17.5–30) and a median length of inotropic treatment of 3 days (IQR 2–4.7). Detailed information on the respiratory and haemodynamic support is provided in Table 1.

On the blood test, the median lymphocyte count was low, as well as the prothrombin time. IL-6 was high at admission. The median for MR-proADM was 1.72 nmol/L (IQR 1.55–2.77), for procalcitonin, 5.7 ng/mL (IQR 1.3–7.77), and for C-reactive protein, 328.2 mg/L (IQR 252.8–351.15). The median values of the NT-proBNP and troponins were elevated in paediatric patients.The results of the blood tests are summarised in Table 2.

All the patients received antibiotic treatment. None of them developed a healthcare-associated infection.

One patient developed coronary aneurisms. During their stay in the paediatric ICU, echocardiograms were performed routinely and showed systolic dysfunction secondary to the shock, but no aneurisms were detected. The coronary aneurisms appeared during the follow-up period, while the patient was in the hospitalisation ward.

The paediatric patients had a median length of stay in the ICU of 6 days (IQR 4.3–7), and they had a total length of stay in the hospital of 12 days (IQR 11–13).

### Comparison between adults and children

While adults were admitted due to ARDS, paediatric patients required admission to the paediatric ICU due to MIS-C (*p* < 0.001). Children had greater inotropic treatment requirements due to the shock, while adults needed it due to the sedation and required lower doses; the differences in the reason for administering inotropic treatment were statistically significant (*p* = 0.005).

There were differences in the blood tests. Children had lower prothrombin time in comparison with adults (*p* = 0.006). Children presented higher values of procalcitonin, C-reactive protein, and MR-proADM (*p* = 0.023, *p* = 0.005, and *p* = 0.017, respectively).

NT-proBNP and troponins were higher in children than in adult patients (*p* = 0.034 and *p* = 0.039, respectively).

## Discussion

The most relevant findings of this study are that children and adults showed different manifestations related to SARS-CoV-2: paediatric patients were admitted due to a shock, with high inotropic requirements, and adults presented with an ARDS, requiring mainly respiratory support. Furthermore, biomarkers as procalcitonin and MR-proADM could be useful to classify and stratify these patients, since their values change depending on the clinical manifestations.

To the best of our knowledge, this is one of the first studies to compare the clinical and analytical differences between critically ill adult and paediatric patients infected with SARS-CoV-2. Moreover, since we are usually a paediatric ICU, adult patients who were sent via referral to our unit were mainly young adults with little or no relevant previous pathologies. This is not a common population, so we think that these results might be interesting for other ICUs.

As far as risk factors, the adult patients in the study had a high rate of obesity (43%). This is relevant, because most of them had no other risk factors. It has already been reported that obesity might be a risk factor for respiratory diseases, as obesity is associated with a decreased expiratory reserve volume, functional capacity, and compliance. Moreover, the inflammatory cytokines associated with obesity might worsen the evolution of these patients when they contract COVID-19 infections [14]. According to other recent articles, obesity, especially when it is related with diabetes and hypertension, might lead to a more serious illness, requiring hospital admission and potentially invasive ventilation [15].

The respiratory treatment of patients with ARDS that needed MV were treated according to the current recommendations for respiratory support [16]: high PEEP, prone position for 16 h a day (when the PaO_2_/FiO_2_ ratio is < 200), and recruitment manoeuvres (when the PaO_2_/FiO_2_ ratio is < 150, if the patient was responsive to PEEP). This strategy was successful, because the evolution of the patients was good overall, except for one partial pulmonary thromboembolism. This patient was already out of the ICU when it happened and was on a high-dose heparin treatment regime (1 mg/kg/12 h). He needed 2 days of NIV support, without the need for inotropic agents, and his clinical evolution was good afterwards. COVID-19 patients have several risk factors for developing pulmonary thromboembolism. The most important one seems to be the presence of an inflammatory state that leads to hypercoagulability, but they also have other risk factors common in critically ill patients, like prolonged immobilisation [17]. An elevated D-dimer value might help to identify these susceptible patients, in order to decide on the best preventive treatment [18, 19]. Heparin treatment is highly recommended in all cases, at least with a prophylactic dose, and this can then be increased depending on the risk factors and the evolution [20]. However, in children there are no specific recommendations, so decisions are taken by extrapolating evidence gathered from adult patients [21].

The respiratory support provided to children was only due to haemodynamic reasons. This is because the clinical features observed in children were completely different. The main problem was shock, not respiratory repercussions. They all needed high doses of inotropic agents, which was a factor that distinguishes them from the adult population. This is consistent with the results of the paediatric study performed by Ramcharan et al., in which all patients had some degree of cardiac dysfunction and their inotropic support requirements were high [22]. In line with this, children with MIS-C had elevated NT-proBNP and troponins values in comparison with the normal values described in paediatrics [23–25], probably because these biomarkers are related to cardiac dysfunction. The paediatric patients had a good evolution overall, but one of them developed coronary aneurysms, which is suggestive of the Kawasaki-like disease [26], that has been described to be related to COVID-19 in children [27].

Taking into account other parametres on the blood test, the paediatric patients showed coagulopathy and an increase in ferritin, lactate dehydrogenase, and D-dimer. There are few reports about critically ill children with COVID-19, but some of them have described similar features, with cardiogenic shock and elevated inflammatory parameters on the blood test [28].

Both paediatric and adult patients had high levels of IL-6. This is consistent with the IL-6 signalling pathway, which seems to be involved in the damage produced by COVID-19. Tocilizumab, which specifically binds soluble and membrane-bound IL-6 receptors, inhibits the binding of IL-6 with its receptor, and consequently inhibits the signal transduction; therefore, it can prevent cytokine release syndrome in this post-infectious inflammatory situation [29]. We would like to remark that, while MR-proADM and procalcitonin were normal in adult patients, they were increased in children, probably indicating an inflammatory situation. C-reactive protein was elevated in all cases, but it was higher overall in paediatric patients. The use of MR-proADM and procalcitonin in children has been previously studied, especially for sepsis, a pathology in which both seem to be useful [30]. Therefore, in this sample these biomarkers might be elevated due to the multisystem inflammatory syndrome. Concretely, MR-proADM is a pro-inflammatory cytokine-induced peptide which has two main effects: vasodilatation and the maintenance of the endothelial barrier [31]. It is a relatively new biomarker that has been proposed as a good tool for detecting invasive infections in paediatric patients [32] and also for stratifying mortality risk in sepsis and cardiogenic shock in adults [33–35]. We believe that its use should be studied in larger samples, since it could be helpful to pinpoint the risk of MIS-C patients.

In this sample, no mortality was recorded, neither in the adults nor in the paediatric cases. In other articles, high mortality rates have been reported (some as high as 61.5% of the patients admitted to the ICU); in most cases, this was related to multiorgan failure and occurred in elderly patients [36]. It is true that our population was younger and healthier, and none of them developed multiorgan failure. However, is it not any less true that, luckily, we were able to provide good care to the patients in terms of the nurse-to-patient ratio, which was 1:2, and physician-to-patient ratio, which was 1:4. Moreover, when the pandemic arrived to Spain, we already had Chinese reports with suggestions regarding patient management, so clearly, being prepared is crucial to improving patient outcomes. Recent works have put on the table the important role of health workers in the management of COVID-19 pandemic and the need for an accurate organisation for the future [37].

We acknowledge several limitations in this study. On the one hand, the sample is relatively small, and on the other hand, the adults are young, so the results might not be able to be extrapolated to other populations. However, we think that it provides valuable information, because this is one of the first studies including both children and young adults in critical condition.

## Conclusions

To sum up, in this sample we observed two completely different entities: adults with ARDS and children with MIS-C. While in the first cohort the main problem was respiratory, with ARDS and high respiratory support requirements, in the second group the problem was haemodynamic in nature, requiring inotropic support to improve the situation. We believe that this could be explained by the fact that the inflammatory response to SARS-CoV-2 seems to be different in adult and paediatric patients, being localised in the lungs or generalised in a multisystem syndrome, respectively. However, this is a small sample, thus further studies with larger samples should be performed in order to confirm these findings.

## Data Availability

The datasets used and/or analysed during the current study are available from the corresponding author on reasonable request.

## Abbreviations

ACE2: Angiotensin-converting enzyme 2
ARDS: Acute respiratory distress syndrome
ICU: Intensive care unit
IL-6: Interleukin-6
MIS-C: Multisystem inflammatory syndrome
MR-proADM: Mid-regional pro-adrenomedullin
MV: Mechanical ventilation
NIV: Non-invasive ventilation
SARS-CoV-2: Severe acute respiratory syndrome coronavirus 2
PEEP: Positive end-expiratory pressure
